# Extradigital Glomus Tumor of the Ankle Associated With Tibial Neuropathy: A Case Report

**DOI:** 10.7759/cureus.117688

**Published:** 2026-10-09

**Authors:** Raphael Zimmermann, Lucas Faria Abrahão-Machado, Flavio Leite Aranha Júnior, Flavio Leite Aranha Neto, Allan Zimmermann

**Affiliations:** 1 Neurosurgery, Hospital São Vicente de Paulo, Jundiaí, BRA; 2 Pathology, Laboratório Bacchi, Botucatu, BRA; 3 Orthopaedics and Trauma, Clínica de Traumatologia e Ortopedia (CTO), Campinas, BRA

**Keywords:** ankle, extradigital glomus tumor, glomangioma, glomus tumor, tibial neuropathy

## Abstract

We report the case of a 26-year-old man with a 14-year history of severe, highly localized pain and allodynia in the right medial retromalleolar region. He had undergone multiple previous medical evaluations and had repeatedly been treated for presumed tendinopathy without meaningful improvement. Magnetic resonance imaging (MRI) of the right ankle demonstrated multiple soft-tissue lesions involving superficial and deep tissue planes around the ankle and hindfoot. Electroneuromyography revealed severe focal axonal neuropathy of the right tibial nerve and its plantar branches. Surgical exploration and excision of the symptomatic lesion were performed. Histopathological examination demonstrated tortuous vascular channels surrounded by uniform round cells, with immunohistochemical positivity for smooth muscle actin and type IV collagen and focal positivity for caldesmon, supporting the diagnosis of a glomus tumor of the glomangioma subtype. The patient experienced immediate and complete resolution of pain and remained asymptomatic at six-month follow-up. This case demonstrates an unusual presentation of an extradigital glomus tumor associated with multiple ankle and hindfoot lesions on MRI and severe focal tibial neuropathy. In patients with persistent, highly localized ankle or foot pain and marked allodynia unexplained by conventional musculoskeletal diagnoses, a glomus tumor may warrant consideration.

## Introduction

Glomus tumors are uncommon perivascular neoplasms arising from modified smooth muscle cells of the glomus body, a specialized neuromyoarterial structure involved in thermoregulation and the regulation of peripheral blood flow [1,2]. They occur predominantly in the distal extremities, particularly in the subungual region, and classically present with severe pain, point tenderness, and hypersensitivity to cold [1,2].

Extradigital glomus tumors are less common and may be difficult to recognize because of their atypical anatomical distribution and less characteristic clinical presentation [1,3]. In a 20-year series of 56 extradigital glomus tumors, Schiefer et al. reported only two lesions involving the ankle and two involving the foot [1]. Similarly, among 11 glomus tumors of the foot reported by Trehan et al., 10 were subungual, and only one was located in the plantar pulp [2]. Diagnostic delay may therefore occur, particularly when these lesions mimic more prevalent musculoskeletal or peripheral nerve disorders [1,3].

Multiple glomus tumors represent an additional diagnostic challenge. In a series of 22 patients with multiple tumors, Anakwenze et al. reported a mean interval of 14.6 years between symptom onset and correct diagnosis, while the complete classical triad was present in only one patient [4]. Magnetic resonance imaging (MRI) is particularly useful in atypical or multifocal presentations because it can assist in lesion localization and demonstrate the extent and multiplicity of disease [1,2,4].

We report an unusual case of a glomus tumor of the glomangioma subtype presenting with approximately 14 years of severe medial retromalleolar pain and allodynia, multiple lesions involving the ankle and hindfoot on MRI, and severe focal axonal neuropathy of the tibial nerve and its plantar branches on electrodiagnostic testing. Surgical excision of the symptomatic lesion resulted in immediate and sustained resolution of pain.

## Case presentation

Clinical presentation

A 26-year-old man presented with a long-standing history of severe pain in the right medial retromalleolar region. The symptoms had begun at approximately 12 years of age and progressively worsened over the following 14 years. He had regularly played soccer since childhood and, during this period, underwent multiple medical evaluations, being repeatedly diagnosed with tendinopathy. Several courses of conservative treatment were attempted without meaningful clinical improvement.

The pain was extremely focal and severe and could be triggered by minimal contact with the affected area. Over time, the increasing intensity of the symptoms substantially limited both sports participation and activities of daily living. Physical examination revealed marked allodynia over a well-defined area of the right medial retromalleolar region, without objective motor deficit.

Imaging findings

MRI of the right ankle was performed without intravenous contrast administration and demonstrated multiple expansile soft-tissue lesions involving both superficial and deep regional connective tissue planes (Figure 1). The largest lesion measured approximately 43 × 28 × 17 mm and was located along the posterolateral margin adjacent to the calcaneus, producing mild remodeling of the adjacent bone without evidence of osseous infiltration. Multiple additional smaller lesions, measuring up to approximately 20 × 14 mm, were predominantly clustered within the medial perimalleolar subcutaneous tissues. The radiological findings were considered nonspecific, with a lesion of neural origin included in the differential diagnosis.

**Figure 1:**
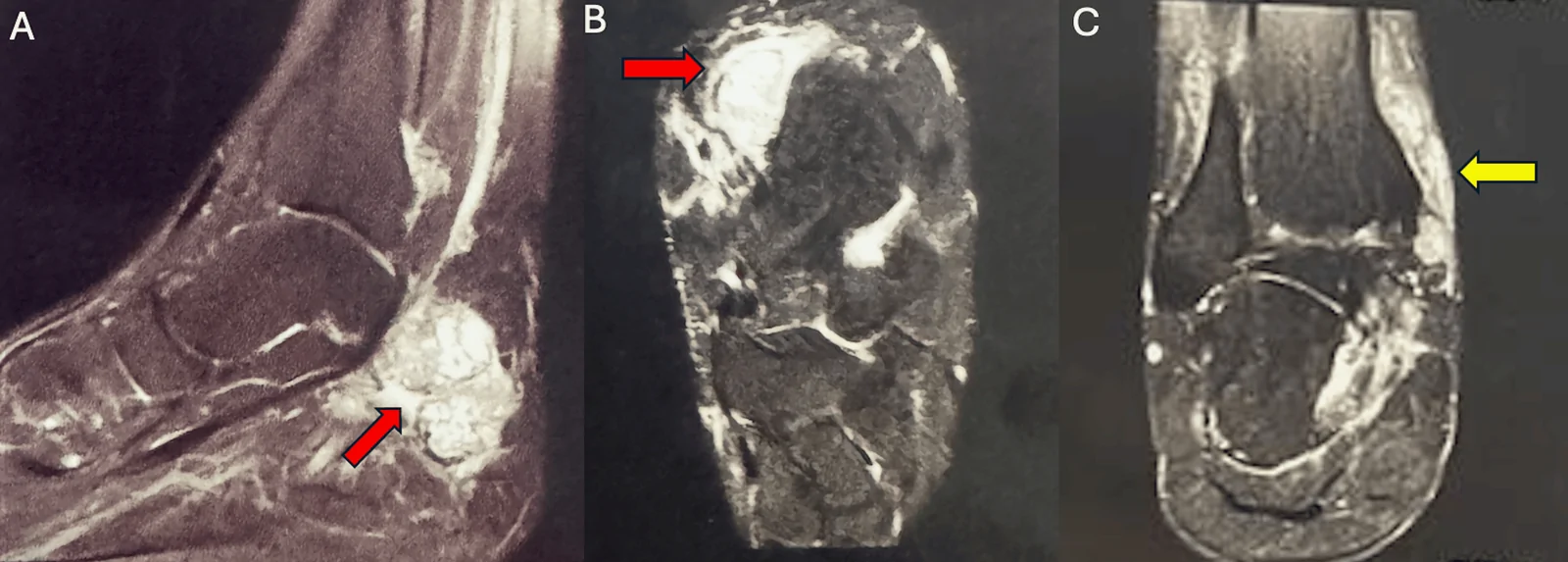
T2-weighted fat-suppressed magnetic resonance imaging of the right ankle demonstrating multiple soft-tissue lesions (A) Sagittal image demonstrating the dominant lesion adjacent to the calcaneus (red arrow). (B) Axial image demonstrating the dominant lesion adjacent to the calcaneus (red arrow). (C) Coronal image demonstrating additional smaller lesions in the medial perimalleolar region (yellow arrow).

Electrophysiological findings

Electroneuromyography demonstrated severe focal axonal neuropathy of the right tibial nerve and its plantar branches. Motor nerve conduction studies showed markedly reduced amplitude of the right tibial motor response, with preserved motor responses in the contralateral limb. Responses from the right medial and lateral plantar nerves were absent. Distal latencies and proximal conduction velocities were preserved.

Needle electromyography of the right abductor digiti minimi demonstrated spontaneous activity at rest and chronic neurogenic motor unit changes with reduced recruitment. Examination of the abductor hallucis could not be performed because of severe regional allodynia. No additional abnormalities were identified in the remaining evaluated peripheral nervous system structures.

Surgical and histopathological findings

Given the severity, prolonged duration, and refractory nature of the symptoms, surgical exploration of the symptomatic right medial retromalleolar region was performed (Figure 2A). A single multinodular lesion involving the soft tissues adjacent to the calcaneus and the medial perimalleolar region was excised en bloc and submitted for histopathological examination (Figure 2B).

**Figure 2:**
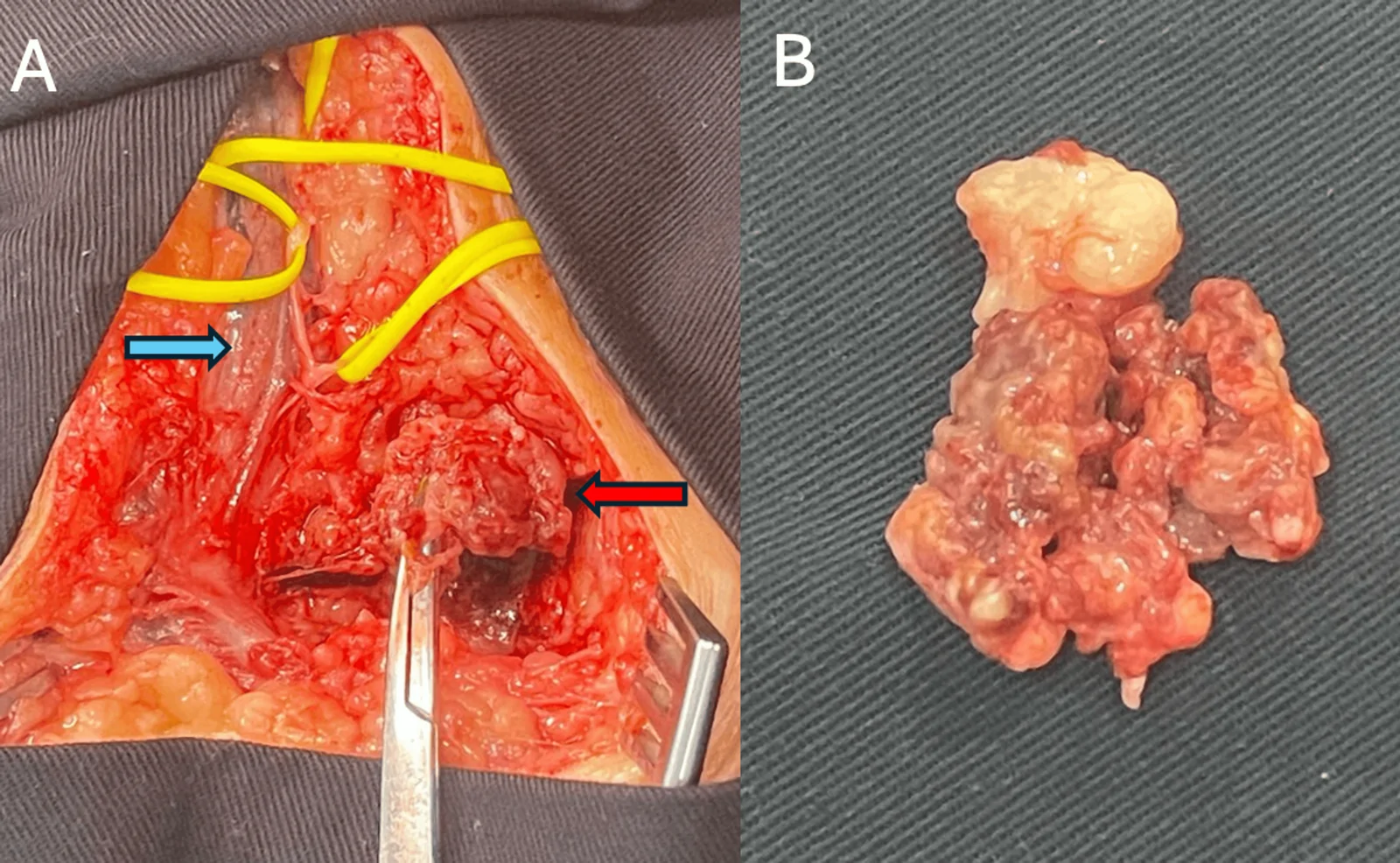
Intraoperative findings and gross appearance of the excised lesion. (A) Intraoperative view of the right medial retromalleolar region demonstrating the lesion (red arrow) and adjacent neural, vascular, and tendinous structures (blue arrow). (B) Gross appearance of the excised multinodular lesion.

Histological examination demonstrated tortuous vascular channels surrounded by relatively uniform round cells with eosinophilic cytoplasm, consistent with glomus cells. Mitotic activity was low (one mitosis per 10 high-power fields), with no necrosis, marked nuclear atypia, or atypical mitotic figures. Immunohistochemical analysis demonstrated positivity for smooth muscle actin and type IV collagen and focal positivity for caldesmon, while CD31 and STAT6 were negative. The overall histopathological and immunohistochemical findings supported the diagnosis of a glomus tumor, glomangioma subtype (Figure 3).

**Figure 3:**
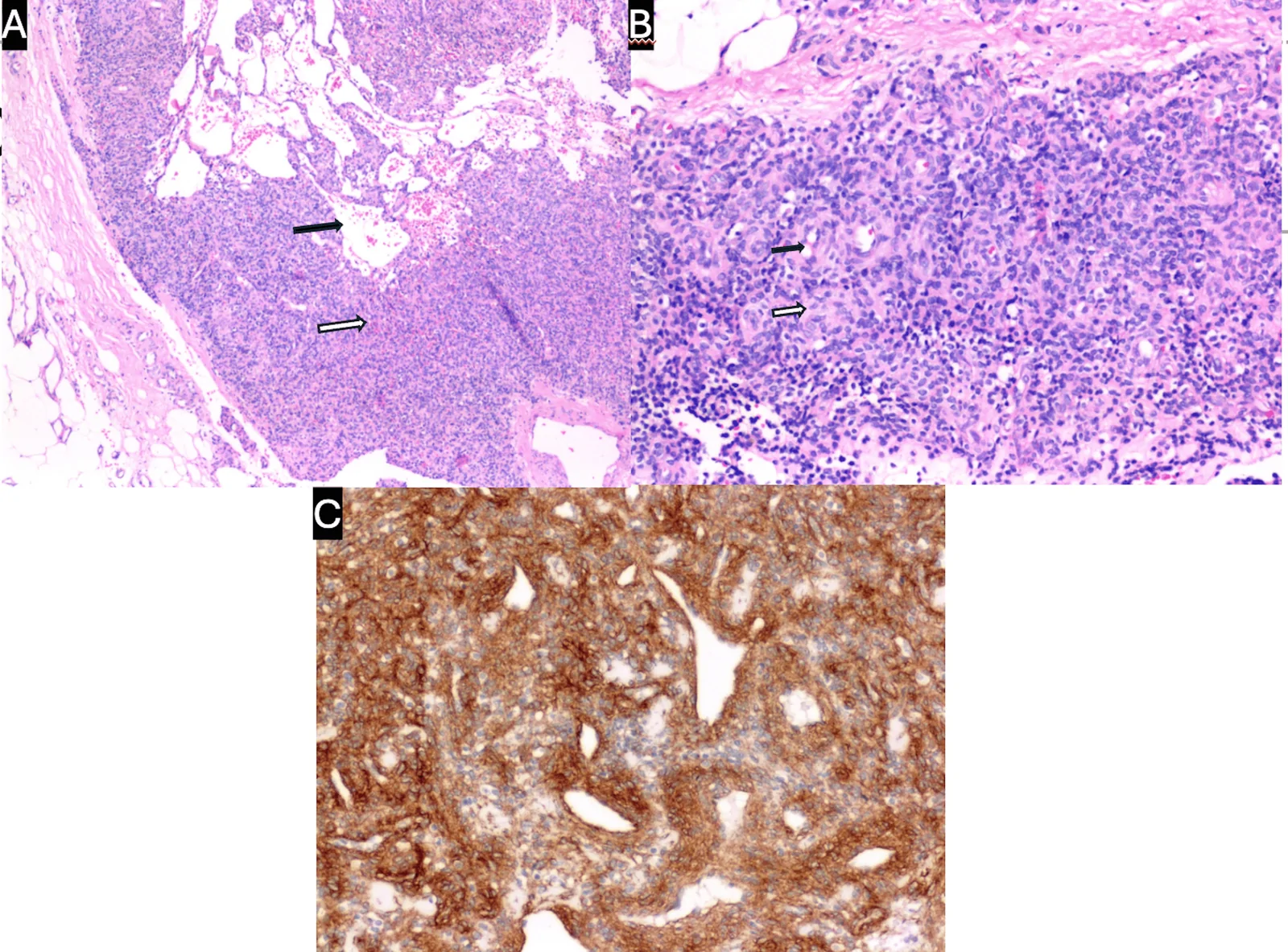
Histopathological and immunohistochemical findings of the glomangioma. (A) Low-power hematoxylin and eosin (H&E) staining demonstrating prominent vascular channels (black arrow) surrounded by cellular areas (white arrow). (B) Higher-power H&E staining demonstrating relatively uniform round cells with eosinophilic cytoplasm (white arrow) arranged around vascular channels (black arrow). (C) Immunohistochemical staining for type IV collagen demonstrating positivity in the tumor cells.

Outcome and follow-up

The patient experienced immediate and complete resolution of the pain following surgical excision. At the six-month follow-up, he remained entirely pain-free, with no recurrence of his preoperative symptoms.

## Discussion

Glomus tumors occur predominantly in the distal extremities, particularly in the subungual region, whereas extradigital tumors have a broader anatomical distribution and are less readily recognized. In the 20-year institutional experience of 56 extradigital glomus tumors reported by Schiefer et al., half occurred in the lower extremity, but only two involved the ankle and two the foot [1]. In the foot-specific series by Trehan et al., 10 of 11 tumors were subungual, and only one was located in the plantar pulp [2]. More recently, De Marco et al. identified 143 patients with extradigital glomus tumors involving the lower extremity in their literature review, further illustrating the heterogeneous distribution and uncommon presentation of these lesions [5].

The prolonged diagnostic course was one of the most notable aspects of the present case. Symptoms began at approximately 12 years of age, and the patient remained undiagnosed for approximately 14 years despite repeated medical evaluations and treatment for presumed tendinopathy. Schiefer et al. reported a mean diagnostic delay of 7.4 years among patients with extradigital tumors [1]. Even more relevant to the present case, Anakwenze et al. reported a mean interval of 14.6 years between symptom onset and correct diagnosis among symptomatic patients with multiple glomus tumors [4]. The striking similarity to the approximately 14-year course in our patient illustrates the diagnostic difficulty that may accompany multiplicity and an atypical anatomical location. Temiz et al. likewise emphasized that extradigital glomus tumors may be misdiagnosed because of their rarity and atypical presentation [3].

The clinical presentation further demonstrates why reliance on the classical triad may be misleading in extradigital disease. Although pain, point tenderness, and cold hypersensitivity are traditionally associated with glomus tumors, Schiefer et al. found pain and localized tenderness in 48 of 56 patients with extradigital tumors, whereas cold hypersensitivity was reported in only one [1]. Among the 22 patients with multiple glomus tumors studied by Anakwenze et al., only one exhibited the complete classical triad [4]. Thus, the absence of documented cold hypersensitivity in the present patient does not argue strongly against the diagnosis. Conversely, his severe focal pain and pronounced allodynia triggered by minimal contact are consistent with the marked tenderness described in extradigital glomus tumors [1,3,4].

Multiplicity is another important feature of this case. Although glomus tumors are usually solitary, multiple tumors constitute a recognized clinical presentation with distinctive characteristics. In the series by Anakwenze et al., 20 of 22 patients had three or more tumors, 90.9% had extremity involvement, and glomangiomas were identified in 15 patients [4]. Lim et al. also described multiple extradigital glomus tumors involving the ankle and foot and highlighted the diagnostic challenge associated with multifocal disease in an atypical anatomical location [6]. In our patient, MRI demonstrated multiple lesions involving superficial and deep tissue planes around the ankle and hindfoot, indicating disease extending beyond the single clinically dominant painful site.

The histological diagnosis of glomangioma is particularly relevant in the setting of multiple lesions. Glomangiomas have a prominent vascular component, with enlarged and irregular vascular spaces surrounded by glomus cells, and have been associated with multiple tumors [4,7]. However, the presence of multiple glomangiomas should not by itself be considered synonymous with glomangiomatosis. Wu et al. described glomangiomatosis as a rare pattern characterized by multiple, deep, and widely distributed lesions with diffuse or infiltrative, angiomatosis-like growth [8]. Because the pathological diagnosis in the present case was specifically glomus tumor, glomangioma subtype, and the available histological findings do not establish a diffuse angiomatosis-like growth pattern, the diagnosis of glomangioma is more appropriate than extrapolating to glomangiomatosis. Previous MRI descriptions of multiple glomus tumors of the foot further demonstrate the value of imaging in defining the distribution and extent of multifocal disease [9].

A particularly unusual feature was the coexistence of severe focal axonal neuropathy of the tibial nerve and its plantar branches. Yoneda et al. reported multiple glomus tumors in a 27-year-old man with medial ankle pain, including one lesion located within the tarsal tunnel at the level of the calcaneus [10]. Their report highlighted glomus tumor as a differential diagnosis in patients presenting with symptoms suggestive of tarsal tunnel syndrome [10]. In the present case, electrodiagnostic testing demonstrated markedly reduced right tibial motor response amplitude, absent medial and lateral plantar responses, and chronic neurogenic changes, providing objective evidence of severe focal axonal tibial neuropathy. This finding distinguishes the present presentation from the previously reported tarsal tunnel case. Nevertheless, anatomical coexistence does not establish causation. Regional compression or irritation by the lesions is a plausible mechanism, but without direct anatomical or postoperative electrophysiological evidence, the neuropathy should be considered associated with, rather than definitively caused by, the glomus tumor.

MRI was particularly valuable because the extent of disease was considerably greater than suggested by the focal symptoms. Schiefer et al. reported that MRI identified the lesion in all 12 patients in whom it was performed [1]. Trehan et al. likewise demonstrated the utility of MRI in patients with glomus tumors of the foot [2], while reports of multiple tumors illustrate its additional value in demonstrating multifocal disease [6,9,10]. In our patient, MRI demonstrated multiple lesions distributed around the ankle and hindfoot rather than a single isolated mass. This distinction has practical implications because not every radiologically identified lesion is necessarily responsible for the dominant clinical symptoms and because knowledge of multiplicity may influence surgical planning and subsequent follow-up.

Histopathological examination remains essential for definitive diagnosis. Glomus tumors characteristically consist of relatively uniform round cells associated with vascular channels and demonstrate smooth-muscle differentiation. In an institutional experience of 138 glomus tumors, Mravic et al. reported α-smooth muscle actin positivity in 99% of evaluated tumors [11]. Folpe et al. similarly demonstrated muscle actin expression in all 40 tumors tested, type IV collagen expression in all eight evaluated tumors, and caldesmon expression in seven of 12 [12]. These findings provide a strong pathological correlate for the present tumor, which was positive for smooth muscle actin and type IV collagen and focally positive for caldesmon. Together with the presence of tortuous vascular channels surrounded by relatively uniform glomus cells, this immunophenotype supports the histopathological diagnosis of glomangioma.

Assessment of biological behavior deserves particular caution in this case. In their analysis of 52 glomus tumors with atypical or malignant features, Folpe et al. identified associations between adverse behavior and parameters including deep location, size greater than 2 cm, and atypical mitotic figures and proposed a classification incorporating these features [12]. These historically proposed criteria highlight the relevance of correlating tumor size and depth with cytological and mitotic features rather than considering any single parameter in isolation. In the present specimen, mitotic activity was low, with one mitosis per 10 high-power fields, and no necrosis, marked nuclear atypia, or atypical mitotic figures were identified. Therefore, the available histological findings do not demonstrate overt cytological features of malignancy, while tumor size and depth warrant clinicopathological correlation, as indicated in the pathological assessment.

Surgical excision is the principal treatment for symptomatic localized glomus tumors and typically results in rapid relief of pain. Trehan et al. reported complete symptom resolution without recurrence among patients with available postoperative follow-up [2]. Yoneda et al. reported complete symptom relief following resection of multiple ankle lesions, including a tumor located within the tarsal tunnel [10]. Temiz et al. likewise observed complete pain resolution and no recurrence at one year in all five patients with extradigital tumors treated surgically [3]. These findings parallel the immediate and complete disappearance of pain in our patient, which persisted at six months. The rapid and sustained postoperative response is consistent with the excised lesion having been the principal source of the longstanding focal pain, although it does not establish that the associated tibial neuropathy itself resolved.

Finally, the multifocal appearance on preoperative MRI has implications for follow-up even after successful excision of the symptomatic multinodular lesion. In patients with multiple glomus tumors, subsequent symptoms may reflect residual disease, a newly symptomatic lesion, development of an additional lesion, or true local recurrence [3,4]. Because postoperative imaging was not available to determine whether any radiologically identified lesions remained after surgery, continued clinical surveillance is reasonable, with repeat imaging guided by recurrent or newly localized symptoms.

Overall, this case demonstrates how an extradigital glomus tumor may remain undiagnosed for many years when it occurs in an atypical location and mimics a common musculoskeletal disorder. The approximately 14-year diagnostic delay closely parallels that reported in patients with multiple glomus tumors [4], while the combination of multifocal ankle and hindfoot lesions, glomangioma histology, and objectively documented severe focal tibial neuropathy represents a distinctive clinical presentation. Glomus tumor should therefore be considered in the differential diagnosis of persistent, highly localized ankle or foot pain associated with marked point tenderness or allodynia, particularly when conventional musculoskeletal diagnoses do not adequately explain the severity or duration of symptoms.

## Conclusions

Extradigital glomus tumors of the ankle and foot may present with prolonged, severe focal pain and remain unrecognized for years, particularly when they mimic more common musculoskeletal disorders. Multifocal disease and the absence of the complete classical clinical triad may further complicate recognition. Glomus tumor should, therefore, be considered in patients with persistent, highly localized pain and marked allodynia when conventional diagnoses fail to adequately explain the clinical presentation.

This case also demonstrates the value of MRI in identifying multifocal disease and histopathological examination in establishing the definitive diagnosis. Severe focal tibial neuropathy was an unusual associated finding, although a direct causal relationship could not be established. Surgical excision of the symptomatic multinodular lesion resulted in immediate and sustained pain resolution. Given the multifocal appearance on preoperative MRI, continued clinical surveillance may be appropriate.
